# Looking Beyond Assumptions to Understand Relationship Dynamics in Bullying

**DOI:** 10.3389/fpsyg.2021.661724

**Published:** 2021-04-20

**Authors:** Faye Mishna, Arija Birze, Andrea Greenblatt, Debra Pepler

**Affiliations:** ^1^Factor-Inwentash Faculty of Social Work, University of Toronto, Toronto, ON, Canada; ^2^Department of Psychology, York University, Toronto, ON, Canada

**Keywords:** cyberbullying, traditional bullying, relationship dynamics, physical bullying, gender, student perspectives, adult perspectives, systems ecological theory

## Abstract

To account for the complex relationships and processes that constitute the phenomenon of bullying, it is critical to understand how students and their parents and teachers conceptualize traditional and cyberbullying. Qualitative data were drawn from a mixed methods longitudinal study on cyberbullying. Semi-structured interviews were held with Canadian students in grades 4, 7, and 10 in a large urban school board, and their parents and teachers. To account for the complexity and interactions of different systems of relationships, the purpose of the current article is to examine how students and their matched parents and teachers understand traditional and cyberbullying. Central to participants' understanding of traditional and cyberbullying was whether they considered bullying to represent harmful relationship dynamics. Three main assumptions emerged as shaping participants' understanding of bullying and appeared to obscure the deep relationship processes in bullying: (a) assumptions of gender in bullying, (b) type of bullying—comparing traditional and cyberbullying, and (c) physical bullying as disconnected from relationship dynamics. It is essential that assessment, education, and prevention and intervention strategies in traditional and cyberbullying be informed by the inherent relationships in bullying and be implemented at multiple levels of relationships and broader social systems.

## Introduction

Bullying is defined as a type of aggression, specifically behavior by an individual or group that is intended to hurt someone (Smith, 2016; Campbell and Bauman, 2018), and that “involves a dynamic interaction between the perpetrator and the victim” (Menesini and Salmivalli, 2017, p. 241). Despite a lack of universal accord on how to define bullying, there is general agreement that bullying is repetitive and entails a power imbalance whereby the perpetrator gains power and the victimized youth loses power, making it difficult for the victimized individual to defend themselves (Smith et al., 1999; Pepler et al., 2010; Smith, 2016). The three main types of traditional bullying victimization that have been delineated and that are encompassed within the overall phenomenon of bullying are physical (e.g., pushing, hitting, kicking), direct verbal (e.g., calling names), and indirect (e.g., spreading rumors) aggression (Lagerspetz et al., 1988; Björkqvist et al., 1992). Also identified is relational aggression (e.g., social exclusion; Crick and Grotpeter, 1995; Espelage et al., 2013), which is considered similar to indirect aggression (Björkqvist, 2018). Corresponding to the definition of traditional bullying, cyberbullying is defined as “an aggressive, intentional act carried out repeatedly by a group or individual, *using electronic forms of contact*, repeatedly and over time against a victim who cannot easily defend him or herself” (Smith et al., 2008, p. 376). The criterion of repetition in cyberbullying is complex, as “a single act by one perpetrator may be repeated many times by others and experienced many times by the victim” (Slonje et al., 2013, p. 27).

The experiences and definitions that youth ascribe to both traditional and cyberbullying do not always align with researchers' definitions, nor with those of parents and teachers (Mishna et al., 2005, 2006; Vaillancourt et al., 2008; Vandebosch and Van Cleemput, 2008). To account for the complex interactions and relationships that constitute the phenomenon of bullying (Bronfenbrenner, 1979), and to ensure development of effective prevention and intervention strategies, it is critical to understand how students and their parents and teachers conceptualize traditional and cyberbullying (Sawyer et al., 2011; Campbell et al., 2019). There is a growing body of research that has compared the perspectives of youth and parents on bullying and cyberbullying (Zeedyk et al., 2014; Midamba and Moreno, 2019), youth and teachers (Giménez-Gualdo et al., 2018; Khanolainen et al., 2020), and parents and teachers (Stockdale et al., 2002; Nguyên and Mark, 2014; Monks et al., 2016; Shea et al., 2016; Campbell et al., 2019). Relatively few studies, however, have explored the perspectives of students and their parents and teachers regarding traditional and cyberbullying (Waasdorp et al., 2011; Cassidy et al., 2013; Compton et al., 2014; Mishna et al., 2020b). The purpose of the current study was to address this gap in the research by exploring how students and their matched parents and teachers understand traditional and cyberbullying in an effort to consider the complexity and interactions of different systems of relationships.

Traditional bullying (Pepler, 2006; Pepler et al., 2010) and cyberbullying (Spears et al., 2009) are considered relationship problems requiring “relationship solutions” (Pepler, 2006, p. 17). The interactive social processes that occur among peers are considered the impetus behind bullying behaviors (Lyng, 2018), whereby bullying is understood as a means through which individuals can meet their needs in the context of their peers or social group (Salmivalli et al., 2010). Specifically, bullying is considered “a form of social power that is exhibited and consolidated in the presence of a relevant social group” (Pepler et al., 2010, p. 470). Rodkin et al. (2015) contend that the focus of prevention and intervention strategies must consequently focus on targeting relationships rather than individual bullying behaviors. To do so it is necessary to examine and understand the problematic aggressive relationship dynamics across and among the perpetrator and victimized youth, the bystanders and the broader networks.

### Ecological Systems Theory

An Ecological systems framework is crucial to understanding and addressing both traditional and cyberbullying, through analysis of the interacting and overlapping factors that influence people at the individual, family, peer and cultural levels (Bronfenbrenner, 1979). According to ecological systems theory, individuals are embedded in and influenced by systems of relationships across the ecological and interconnected contexts, which individuals, in turn, influence (Bronfenbrenner and Morris, 2007; Wang et al., 2016). As such, children's social-emotional development at school is affected not only by children's relationships with their teachers and their peers, but also by the connections between these relationships as well as the other levels of social ecology, all of which are seen as contributing to social behavioral patterns (Pepler et al., 2004; O'Moore and Minton, 2005). For example, teacher–student relationships are a central element of the social ecology of schools and can contribute to adaptive social–emotional development of students (Wang et al., 2016). The nature of student–teacher relationships may be protective against distressing peer victimization (Sulkowski and Simmons, 2018) or on the contrary may contribute to students' problematic relationship patterns (Mishna et al., 2008; Wang et al., 2016).

To understand traditional bullying and cyberbullying therefore, taken into consideration are factors that shape youth's vulnerability to involvement in traditional and cyberbullying, as victimized and/or perpetrator, at multiple levels. These levels include emotional and cognitive development, family dynamics and situation, peer interactions, and cultural and societal conditions (Bronfenbrenner, 1992; Bronfenbrenner and Morris, 2007; Espelage, 2014; Cross et al., 2015). An ecological systems framework focuses on the notion that relationships across all levels of the ecological system are interrelated and not independent. In recognition of the “seamless online/offline social context of young people's lives and the means by which they engage with others in online contexts” (Cross et al., 2015, p. 110), recent additions to this framework extend a child or youth's social ecology of home, school and community environments to include the cyber world (Johnson, 2010). This cyber addition is critical given the unique social context, transformative nature, and central role online interactions now have in the social lives of youth (Nesi et al., 2018).

The aim of the current study was to expand the limited body of research that examines how traditional and cyberbullying are understood by youth, parents and teachers, who represent three critical systems of relationships in the ecological context of bullying. To develop effective prevention and intervention strategies, it is essential to understand how both youth and adults conceptualize the nature and impact of bullying (Vaillancourt et al., 2008; Vandebosch and Van Cleemput, 2008).

## Materials and Methods

### Baseline Study Sample

Data for the present study were drawn from the qualitative component of a 3-year mixed methods study, in which we investigated how youth and their parents and teachers perceived traditional bullying and cyberbullying. Stratified random sampling was used to select schools (*n* = 19) in a large Canadian urban school board. To ensure ethnocultural and socioeconomic diversity, the schools were classified according to a school board index based on external barriers to student achievement and were then stratified into three categories of need (i.e., low, medium, high; Mishna et al., 2016).

Neighborhood-level census data used to develop the school board's index included parental income and education levels, ratio of households receiving social assistance, and ratio of single parent families (TDSB, 2014). We chose stratified random sampling to ensure representation of ethno-cultural and socioeconomic diversity, factors that potentially impact access to information and communication technology (ICTs), experiences of cyberbullying, and the manifestation of negative outcomes (Lenhart et al., 2015; Steeves, 2015). The total sample comprised students in grades 4 (*n* = 160), 7 (*n* = 243), and 10 (*n* = 267), as well as their parents (*n* = 246) and teachers (*n* = 103). In year three of the study, 10 additional schools were recruited for participation to follow students transitioning from elementary/middle school to middle/secondary school. A total of 29 schools therefore participated in the study. The students, parents and teachers all completed quantitative questionnaire packages. Quantitative data were collected from students and parents in each year of the study. Teachers participated in year one only, as they changed every year. In addition to this survey data, a series of 137 qualitative semi-structured interviews were conducted with a selection of students, their parents and teachers, which is outlined in the following section.

### Current Study Sample

Students were purposively selected from the total sample to participate in interviews. Students were invited to take part based on gender, grade, and level of school need. Student involvement in bullying/cyberbullying as a victim, perpetrator, witness, or non-participant, was assessed based on their self-reports in the survey. We purposively selected students according to their category of involvement. In year one, semi-structured interviews were conducted with 57 students (20 fourth grade students, 21 seventh grade students, and 16 tenth grade students), 50 parents, and 30 teachers. In year three, interviews were conducted with 43 and 29 of the same students and parents, respectively. Teachers were interviewed in year one only, and no interviews were held in year two. Some teachers gave responses for more than 1 student. Participants received a $10 gift card at each interview. Student quotations are identified by grade at the time and study year of the interview, as well as by gender (Mishna et al., 2016, 2020a,b). Ethics approval was received from the University Research Ethics Board and the School Board External Research Review Committee, and parental consent and student assent were obtained.

### Sample Demographics

Of the 57 students who consented to participate in the qualitative component of the study in year 1, 20 students were in grade four, 21 in grade seven and 16 were in grade 10, ranging in age from 9 to 16. Sixty-one percent were girls and 39% were boys. Their identified race/ethnicity was: 30% White; 32% Asian; 5% Black; 25% Other/Mixed; 3% did not know; and 5% missing. Of the 38 parents who completed the demographic questionnaire in year 1, 82% identified as female and 18% as male. Thirty-three percent were born in Canada, 16% in Pakistan, and 8% in China. Over 56% spoke English at home while 14% spoke Urdu, and 87% self-identified as Canadian. Fifty percent had completed college, university, or held a professional degree; 28% had a household income of $39,999 or lower and 25% $100,000 or higher. Of the 14 teachers who completed the demographic questionnaire, 43% identified as female and 57% as male; 79% identified as White, 14% as Asian and 7% as Middle Eastern. While 86% were born in Canada, all self-identified as Canadian (Mishna et al., 2016, 2020a,b).

### Data Collection

Individual interviews, lasting between 30 and 90 min, were conducted by 10–15 trained research assistants, primarily Master of Social Work students or graduates. The students and teachers were interviewed in a private location in their schools and parents were interviewed in person or over the telephone, based on their preference. The interview guide was informed by a thorough review of the literature including previous interview guides and the team's research and practice experience (Mishna et al., 2016, 2020a,b). The interview guide encompassed five broad areas. These included: (1) cyber world context (e.g., can you tell me about your use of cyber technology?); (2) bullying/cyberbullying context (e.g., do you think cyberbullying is a normal part of growing up?); (3) Motivations (e.g., what do you think kids get cyberbullied about?); (4) differences between cyberbullying and face-to-face bullying (e.g., do you think that being cyberbullied is different from being bullied face-to-face?); and (5) Getting help (e.g., what stops young people from getting help?). The interviews were audio-recorded and professionally transcribed. Questions included how traditional bullying /cyberbullying was defined, perceived motivations for traditional bullying/cyberbullying, experiences with technology and bullying, and whether participants considered traditional bullying/cyberbullying a problem (Mishna et al., 2016, 2020a,b).

### Data Analysis

Using a grounded theory inquiry, data were concurrently analyzed and theorized in a reciprocal process of constant comparison (Corbin and Strauss, 2008; Birks and Mills, 2015). This iterative process allowed the team to use initial interview data and theoretical categories to inform, refine and focus, subsequent interview guides and data collection (Charmaz, 2014). The team members individually coded a portion of interviews to establish preliminary analytic focuses, and inductively identify preliminary themes. As a group, the team members then examined all coded interviews, which revealed overall coding agreement. Differences were discussed and revised, based on consensus. Emerging categories were developed and expanded. Axial coding promoted connections within and between categories and subcategories and enabled synthesis and explanation (Corbin and Strauss, 2008; Charmaz, 2014; Birks and Mills, 2015). Numerous preliminary codes were identified based on emerging themes that were generated and discussed. Following this, holistic “middle-order” coding enabled us to condense the number of codes (Saldaña, 2015). Through this iterative process of open, holistic, and focused coding, key themes emerged related to the understanding of traditional and cyberbullying according to the perspectives of the students, parents, and teachers.

Measures were employed to ensure trustworthiness and authenticity. Prolonged engagement over the 3 years of the study ensured thick descriptions of the youth and adult narratives (Lietz and Zayas, 2010). Rigor was established through documentation for auditing purposes (Padgett, 2008). Trustworthiness and transferability were further ensured through reflexive journaling, bracketing, and dense descriptions (Corbin and Strauss, 2008).

## Results

Analysis of the interviews with students, parents and teachers revealed an overarching theme, *the relationship dynamics of bullying*. This overall theme of whether participants understood bullying as occurring in the context of relationships, encompassed three interconnected sub-themes: (a) assumptions of gender in bullying, (b) type of bullying—comparing traditional bullying and cyberbullying, and (c) physical bullying as disconnected from relationship dynamics.

### Overarching Theme: The Relationship Dynamics of Bullying

Bullying arises out of power dynamics in a relationship, typically with repeated interactions that consolidate the power differential and shape one's sense of belonging (Smith et al., 1999; Pepler et al., 2010; Smith, 2016). In discussing traditional bullying and cyberbullying behaviors and episodes, it emerged that the ways that the students, parents, and teachers understood bullying appeared to be largely based on their assumptions, which shaped whether they considered bullying to occur in the context of relationships.

### Sub-theme a: Assumptions of Gender in Bullying

Analysis of the interviews revealed that the students as well as their parents and teachers tended to characterize boys' and girls' bullying and cyberbullying behaviors in ways that appear consistent with dominant gender stereotypes and norms. Generally, neither the students nor the adults appeared aware of the influence of these gendered stereotypes and norms on their understanding. Participant accounts of the traditional bullying and cyberbullying behavior indicated that while boys and girls were often described as prone to engage in different forms of bullying behaviors, both boys and girls were involved in ongoing, repetitive bullying episodes. Analysis suggested however, that the assumptions participants conveyed about gendered behavior seemed to shape whether they understood specific traditional bullying and cyberbullying incidents as complex social relationship problems (Pepler, 2006). For example, one girl stated that girls “usually talk. We usually like to make groups and attack each other.” She explained that unlike girls, boys “just shout out loud in the field and fight” (Grade 4 girl 673, year 3). Based on such gendered assumptions, participants tended to differentiate girls' bullying as complex and connected. In contrast they often described boys' bullying as untethered acts of aggression—coming out of nowhere, just arising, then quickly disappearing—and not part of an ongoing relationship dynamic. Participants rarely questioned these assumptions.

Youth and adult participants routinely portrayed boys as “chill,” not dwelling on issues, forgetting bullying episodes quickly, not making a big deal and not involved in bullying as a “big thing.” Illustrative of this characterization, one parent explained that with boys, “there are little things like that going on, but I don't think it's the really vicious stuff. I think it's just more the teasing side of stuff, and I don't think it's constantly in a kid's face” (Parent of grade 4 boy 020, year 3). In contrast, girls were typically portrayed by the youth and adult participants alike as “nasty,” more likely to “hold grudges,” “overly sensitive,” and “complicated.” For instance, in identifying with her daughter's bullying experiences, a parent said, “I can definitely sympathize with her. I haven't really had that much experience with it with my son, but I know girls can be very nasty. I remember going through my own nasty situations with girlfriends when I was growing up” (Parent of grade 7 girl 009, year 3). Another parent likewise maintained, “there is a tendency for I guess women or girls to be more vindictive” (Parent of grade 7 boy 377, year 3). Similarly, a grade 10 girl explained,

Girls are just different personalities. I think they're easier to get upset over things. The little things bug them, “you didn't phone me when you said, you didn't wait for me, whatever, you talked to somebody else and didn't include me.” Boys, I know they don't care about those things, the girls care about all those little things. (Grade 10 girl 896, year 1).

Participants consistently delineated a profound difference in the ways boys and girls interact in their relationships. According to many participants, boys “scrap it out” and “then it's over,” whereas girls “hold grudges” and it “goes on forever.” In describing experiences with her daughter, one parent stated:

Girls, at least I found with [009's] situation, once they find something that bothers you or rubs you the wrong way, they just dig, and they dig and they dig and it turns in to be a real cat fight that goes on forever. Whereas guys, if something makes guys mad, they punch each other and then it's over with and the next day, like they don't seem to hold any grudges and it doesn't seem to last forever like it does with girls (Parent of grade 7 girl 009, year 1).

In comparing boys and girls, a teacher similarly described girls as “catty” and their bullying as “very dramatic when it doesn't need to be.” This teacher believed that because the situation among girls “gets blown out of proportion” and becomes “this big thing,” girls “do get hurt.” In contrast, this teacher declared, “boys don't really bully each other to that point.” Despite elaborating that boys “mostly bully boys when they know that they can have more power over somebody that they feel has less power than them,” the teacher made no mention of possible harm or effects of the bullying among boys (Teacher of grade 7 girl 501).

While participants typically characterized girls and boys based on gendered personality stereotypes when describing their bullying behaviors, there were some exceptions. Rather than emphasizing assumed inherent gendered personality traits as driving bullying behavior, a few participants referred to the relationship dynamics of bullying or contemplated the effects of gendered stereotypes and norms. For example, one teacher who interpreted relationship dynamics as intrinsic to bullying commented, “it's basically four little girls who each want to be queen bee right now, and so that changes from day to day, who has power over someone else. So, there's a lot of exclusion tactics…” (Teacher of grade 4 girls 314 and 312). After stating that girls and women are “more vindictive,” whereas boys and men have “your spat, you get over it, and you move on,” one mother questioned these assumptions: “I don't know how much of it is just media driven because I guess the victims that we see on the news, at least in Canada, have been girls, right?… but that doesn't say that boys aren't also being bullied” (Parent of grade 7 boy 377, year 3).

A girl who commented that girls bully each other because of appearance spoke up in praise of boys, stating, “that's the thing I like about guys because usually they don't tend to worry about those things…. They're proud of themselves, and they don't pick on other people. They're good with what they have.” Like the parent above, after making these comments, the girl contemplated the origins of these differences between boys and girls: “I think it's from when we were little because those Barbie dolls are super skinny. We wanted to have blonde hair, blue eyes, and be like Barbie or something like that. I think it's just how maybe we were raised” (Grade 4 girl 312, year 3). Another girl who declared that cyberbullying occurred with equal frequency among boys and girls commented that it wasn't “a big thing” for boys whereas girls, “would show it off more, be like oh yah, blah, blah, blah.” Rather than concluding that this difference indicated that cyberbullying was not a big deal for boys, however, she alluded to the influence of dominant gender norms:

Guys kind of hide it in more….I think mostly if they're being bullied because they don't want to show that they're weak because guys tend to be, they think that they're very strong, kind of thing, so I don't think they would show it as much. Girls kind of like the vulnerable look, so I think girls tell, more than guys do (Grade 7 girl 421, year 3).

The analyses suggest that participants typically viewed boys and girls as engaging in bullying behaviors in highly divergent ways. Participants tended to focus on the gendered personality assumptions of girls and boys rather than contextualize their interactions and behaviors as occurring in complex relationships influenced by power relations and societal norms.

### Sub-theme b: Type of Bullying—Comparing Traditional Bullying and Cyberbullying

The participants' responses revealed both similarities and differences in how they understood traditional bullying and cyberbullying. In comparing types of bullying with respect to ease in which to engage and which type has more severe and lasting impacts, it emerged that participants' assumptions seemed to preclude them from acknowledging the contextual aspects of bullying. Similarly, their assumptions regarding the roles of victims, perpetrators, and bystanders appeared to often prevent participants from explicitly recognizing the inherent power and relationship dynamics of bullying. The following statement by a teacher who did not take into account two central components of bullying, which are the intent to cause harm and the power dynamics, illustrates the inconsistency in how bullying relationship dynamics were considered: “I think it's more difficult to confront someone face-to-face, and it's very easy to hide behind a computer and do those very same things that you wouldn't do if you had to face the person that you're bullying” (Teacher of grade 4 girl 314).

#### Engaging in Cyberbullying Is Easier

The students, their parents, and teachers tended to consider cyberbullying easier to engage in than traditional bullying. A central reason given by participants for holding this view was that in cyberbullying, “you're under the cloak of darkness, and when you are sending that email, text, or writing on somebody's wall, there's that disconnect” (Parent of grade 4 girl 312, year 1). As explained by a teacher, “when they can't see the person's face, if they can't see the hurt, they can't see whatever it happens to be, then I think that sometimes they do it without thinking” (Teacher of grade 7 boy 145). A grade 7 girl similarly remarked that youth who bully can “say a lot more” online or that they can “say as many things as they want.” In comparing this ease with the relative difficulty of traditional bullying, this student explained that because in traditional bullying the perpetrator can “see how the person feels,” they “can't really say much” because “it takes them down probably just a little bit. They will say things, but not as hurtful” (Grade 7 girl 421, year 1).

Several participants posited that unlike in cyberbullying, being able to see the impact of bullying victimization in traditional bullying serves to discourage youth from persisting with bullying behaviors. A teacher who reflected this viewpoint explained that more of the youth “who have a conscience will realize “oh my gosh, this is a real person I'm doing this to and it's hurting them,” and they'll stop.” Conversely, this teacher believed that “cyberbullying will get worse because they can't see it when they're on a computer at home, in isolation. They can't see the effects” (Teacher of grade 7 boy 145). Likewise, a parent who considered cyberbullying more conducive to bystanders joining in, elaborated, “you wouldn't surround somebody and start kicking them because you know that you're causing pain. But, if you're just adding another comment to what somebody else has already added, it might not seem as bad” (Parent of grade 4 boy 341, year 1).

Some participants went so far as to suggest that a consequence of not seeing the impact of cyberbullying on the victim's face, is that it takes more “courage” to engage in traditional bullying. In contrast, they considered cyberbullying to be an act of weakness. As one parent explained, “if you're cowardly or weak, you still, mean people come in all shapes and sizes and characters. So, I think the fact that you can do it virtually makes it easier…” (Parent of grade 4 girl 312, year 3). Another parent contended, “anybody who cyber bullies…has no backbone because they don't have to worry about the confrontation or the message they're going to receive. Yeah, they're going to get a typed message in return, but there's nothing there” (Parent of grade 10 girl 812, year 3). Similarly, a teacher who stated that it is significantly more difficult to engage in traditional bullying claimed, “you need to be more courageous. You need to have more guts. It takes a lot more integrity actually to do it in front of somebody's face” (Teacher of grade 4 boy 020). In talking about the role of confidence in determining whether someone would engage in traditional bullying or cyberbullying behavior, a student similarly suggested, “they'd be more likely to insult someone or harass someone in person because they're not scared of what the person could do back to them” (Grade 7 girl 009, year 3).

Participants often reflected on which type of bullying they felt had more detrimental effects. Analysis revealed that participants' divergent perspectives on the effects of traditional bullying and cyberbullying encompassed three main views: (1) cyberbullying is worse because of its enduring evidence and effects, (2) traditional bullying is worse because of the potential for physical as well as emotional harms, and (3) despite substantive differences between traditional bullying and cyberbullying, they are essentially equivalent as they have the same effects.

#### Experiencing Cyberbullying Is Worse

Many participants thought cyberbullying was worse for various reasons, including the potentially limitless number of bystanders in contrast to the limited number in traditional bullying. One teacher commented that there are “so many more bystanders to what's going on, so the humiliation is multifold” (Teacher of 2 grade 7 girls 140 and 141). Several participants emphasized that the effects of bullying are magnified by the possible exponential spread and permanence of cyberbullying. One student judged cyberbullying to be worse than physical bullying, “because they say words and words can get in your head forever” (Grade 4 boy 341, year 1). A parent who concurred that cyberbullying causes more harm, qualified her response by underscoring that all bullying must be taken seriously. When contrasting traditional bullying and cyberbullying she remarked that traditional bullying comes to an end, whereas in cyberbullying, “the pain is prolonged and it hurts more,” there is “more time to chew on what was said to you and then you just kind of get into this infinite loop of why, why, why?” (Parent of grade 4 girl 314, year 3).

#### Experiencing Traditional Bullying Is Worse

Participants who considered traditional bullying to be more serious than cyberbullying generally believed this was due to the possibility of experiencing physical hurt, which would amplify the emotional hurt. Of note, this view is somewhat inconsistent with the notion that physical bullying isn't as bad because bruises and other physical injuries heal. A student noted that in cyberbullying, “you can just say a bunch of mean stuff, that's all you can do.” This student went on to explain that traditional bullying is more serious because, “you can get into physical fights, that's more dangerous because you can get hurt with that. You're also hurt while you're getting cyberbullied, but you're just not hurt as much” (Grade 7 boy 145, year 1). Another student who likewise highlighted the possibility of being “punched” and “kicked,” claimed, “but if it's on the Internet, just with words, then you would forget about it at some point.” This participant added that whereas words on the Internet can be deleted, “it would be stuck in your head if you listened to it” (Grade 4 girl 347, year 1).

Other participants regarded traditional bullying worse because of the intensity of the interactions. One student for example considered cyberbullying less embarrassing, “because there's obviously going to be bystanders and the bystanders in your face, if they see them they're going to spread rumors and on the internet nobody cares” (Grade 7 boy 154, year 1). A grade 7 teacher similarly remarked that a victimized youth feels “worse when it actually happens in real life. If someone just said, on the internet, we're going to exclude you, and then it never happened, I don't think that's as powerful, I don't think that's as brutal or powerful” (Teacher of grade 7 boy 106). A parent also held the view that while there are parallels between traditional bullying and cyberbullying, the physical presence of another person made traditional bullying a more intense and potentially harmful experience:

I just think that when somebody is right into your physical body…you can see them and your energy is there with their energy, and they're saying things to you or throwing things, or whatever they're doing…your physical body could be hurt as well…But, it's just different [cyberbullying], because they can't physically hurt you as easy when they're not in the room with you. Well, they are in the room, but not able to throw something at you, except for words and that kind of thing. Which is very upsetting, I'm not minimalizing the effects of that, but I'm just saying that it can feel even more, to me, invasive if somebody is there right beside you (Parent of grade 7 girl 374, year 3).

#### Traditional Bullying and Cyberbullying Are Essentially Equivalent

A number of student and adult participants judged traditional bullying and cyberbullying to be equivalent. While some of these participants initially noted the equivalence, others initially indicated that one type was more serious, and it was only as they reflected in the interview that they came to a different understanding.

One student definitively asserted that cyberbullying and traditional bullying “both hurt. They're both just as bad.” She elaborated that with “physical bullying in real life, your peers and friends will see it, and you can't get away from it,” whereas, “cyberbullying affects you mentally a lot.” She concluded, “I don't know but they're both really bad, and that's the end of my story” (Grade 10 girl 290, year 1). Some participants considered traditional bullying and cyberbullying to be equivalent, because “in both cases, the victim is being harassed, the victim is hurt” (Grade 10 girl 896, year 1). This girl elaborated that while physical hurt was not a threat in cyberbullying unlike traditional bullying, “at the same time, cyberbullying could go from like people bullying you on Twitter to one day seeing you face-to-face and it could become worse like that. But yeah, it is the same, to me” (Grade 10 girl 896, year 1).

Despite initially saying that traditional bullying was more serious because it might lead to physical bullying, a student reflected on the harms of different types of bullying: “Like cyberbullying can affect them emotionally, physical bullying can affect them physically and verbally can affect them by mentally” (Grade 7 male 310, year 1). Notwithstanding that traditional bullying has the potential to become physically aggressive/violent, students and adults alike recognized the extent to which youth depend on the cyber world, which one participant termed the “playground in their world.” As explained by a teacher, “even though it [cyberbullying] probably is not as violent, it could have just as much of an effect in terms of intimidation and exclusion” (Teacher of grade 7 girls 414 and 421).

In comparing traditional bullying and cyberbullying, participants often appeared to make assumptions about the complex relationship dynamics among perpetrators, bystanders and victimized youth. Analysis of the transcripts revealed, however, that students, parents and teachers seemed unaware of their assumptions. Moreover, many participants made contradictory statements within their own narratives, which attests to the complexity of bullying. For example, several participants concluded that it would be harder for the perpetrator or bystander to sustain their bullying roles in person because of seeing the impact. In stating that bullying perpetration and witnessing is harder to sustain when seeing the victimized youth, there is an implicit flagging of the salience of complex relationship dynamics in bullying. On the other hand, when describing cyberbullying as easier in which to engage, there is a minimization of the relationship dynamics of cyberbullying. Yet, while saying that the effects of cyberbullying are worse because of the potential infinite number of bystanders, participant discussions again draw attention to the importance of the relationship dynamics in bullying. Thus, at different times and in different ways, both implicitly and explicitly, participants relegated the complex relationship dynamics in bullying to the margins.

### Sub-theme c: Physical Bullying as Disconnected From Relationship Dynamics

In comparing types of bullying, participants tended to differentiate physical bullying and cyberbullying. This distinction is evident in a student's comparison in which he stated, “face-to-face is like you hurt them like outside, in the body, but when it comes to technology, it hurts like inside, in your heart” (Grade 10 boy 641, year 1). Thus, a striking theme that emerged was characterization of physical bullying as not occurring within relationship dynamics (Pepler, 2006). Several students and adults distinguished physical bullying based on the associated visible hurt and injuries and focused on the fact that injuries heal. In so doing, these participants did not appear to consider the complex relationship dynamics of physical bullying. A grade 10 boy's statement exemplified this understanding: “physical is short-term, like you just get hurt physically, and you'll heal in a few days but, if it's mentally, it might stay for a long term and maybe might have more effect” (Grade 10 boy 211, year 1). Gender figured prominently in participants' conceptualization and descriptions. While the students and adults spoke about boys' physical bullying as unrelated to relationship dynamics, participants discussed traditional bullying and cyberbullying among girls as entrenched in complex relationship dynamics. A parent stated, “Boys I think if there's a skirmish it's physical and then it's done. Girls…they're easier to get upset… Little things bug them more” (Parent of grade 7 girl 504, year 1).

Such thinking seemed to contribute to participants viewing physical bullying as defined by the actual hurt or injury and to discounting the relationship context in which the hurt or injury occurred. This view is exemplified by a girl who commented, “I think bullying, it's bad when someone beats you actually. But I think it's bad or maybe even worse when you're just abused emotionally because it's something that's not going to go away easily” (Grade 10 girl 640, year 1). Missing from such narratives is mention or acknowledgment of the relationship dynamics in which a person(s) intentionally caused the injury by physically hurting the victimized youth. Accordingly, participants portrayed these episodes and their effects as over once the physical wounds healed. This sentiment is evident in another girl's statement that because physical injury heals, “as long as they use no words you're just going to get better.” In contrast, she noted that in cyberbullying not only is there a record but, “sometimes you just keep the thoughts mentally, if they didn't hurt you physically, they really hurt inside. Just to know that they are thoughts and not actually like hits they still hurt even more” (Grade 7 girl 501, year 1). Concurring with this view, a parent maintained,

online you could read it over and over again and the hurt just gets worse and worse. If someone hurt me physically, I see the bruise and I have an image of someone hitting me, but I think it's different when I'm reading again and again…the impact is more I think (Parent of grade 4 girl 314, year 1).

Not all participants, however, relayed the view of physical bullying as devoid of a relationship context. Some participants acknowledged the relationship context of bullying, albeit relationships that may be unhealthy and undesirable. As one girl explained, “for girls it's more of talking badly about someone behind their back or even to their face. But for guys, it's more physical. If a guy didn't like another guy, he wouldn't talk about him. He'd probably beat him up” (Grade 7 girl 009, year 3). While this girl's statement illustrates active rejection used by both boys and girls, it suggests different dominant strategies and displays, that nonetheless, arise from underlying relationship dynamics.

A teacher similarly acknowledged the relationship dynamics in both traditional bullying and cyberbullying:

I think with the cyberbullying, it's more an emotional thing, it affects you, you read about this and what's going on. Whereas, being face-to-face with the bully, there's a physical threat of it all and there is also the emotional and the fear, it's present in that situation (Teacher of Grade 7 girl 139 and Grade 7 boy 154).

In describing the effects on victimized youth, one boy alluded to the power imbalance in both physical bullying and cyberbullying, stating,

I believe the victim is largely similar simply because of like when the bullying, how it affects their state of mind. Because like they'll feel more weak, scared, and they'll be like nervous, anxious, a tad paranoid, so yeah, they'll be like a lot more, they'll feel a lot more weak, regardless of the method of bullying (Grade 7 boy 377, Year 1).

## Discussion

Findings revealed that central to student, parent and teacher perspectives of traditional bullying and cyberbullying was whether they understood bullying as representing harmful relationship dynamics. Analyses of the interviews revealed this as an overarching theme, *the relationship dynamics of bullying*. This overall theme of whether participants understood bullying dynamics as occurring in the context of relationships, encompassed three interconnected sub-themes: (a) assumptions of gender in bullying; (b) type of bullying—comparing traditional bullying and cyberbullying; and (c) physical bullying as disconnected from relationship dynamics. The overarching theme and sub-themes highlight how participant assumptions in some ways obscure the deep relationship processes in bullying that contribute to the harm. As participants discussed their understanding of bullying, they sometimes reconsidered their understanding and the complexity of bullying, which brought them to different conclusions.

### Assumptions of Gender in Bullying

The participants overwhelmingly concurred that boys and girls differ significantly in their bullying involvement and experiences. When discussing bullying, the student and adult participants overwhelmingly characterized boys through such descriptors as “chill,” not bothered by “little things,” not dwelling on issues or holding grudges, and not involved in bullying as a “big thing.” In contrast, they portrayed girls as “nasty,” “catty,” “overly sensitive,” and bothered by “little things,” with a tendency to “hold grudges” and “not let things go.” In their descriptors of how boys and girls bully, the participants implied that addressing bullying among girls was complex and ongoing whereas it was easier to address bullying among boys. Eriksen and Lyng (2018) similarly found such characterization among teachers, who described boys as “simpler” and girls as doing “meaner things” (p. 400). Moreover, the teachers clearly delineated boys' and girls' bullying behaviors, asserting, “Boys resolve the conflict there and then. They are more peaceful” (Eriksen and Lyng, 2018, p. 400).

These assumptions appeared to preclude participants from discussing bullying in a manner that acknowledged the relationship dynamics integral to bullying. This process echoes previous research that examined gendered assumptions and narratives in bullying among students. Ringrose and Renold (2010) demonstrated that heteronormative discourses served to render both masculinized and femininized bullying behaviors as something integral to being either a boy or girl, respectively. Normalizing or naturalizing bullying behaviors as inherently due to one's gender and thus fixed, diffuses responsibility for addressing everyday gender-based violence and aggression (Ringrose and Renold, 2010) and “ignores the power relations in which the bullying occurs” (Horton, 2011, p. 271). Indeed, the researchers noted that these “everyday gender performances are frequently passed over by staff and pupils as ‘natural’” (Ringrose and Renold, 2010, p. 573). Reflecting dominant patriarchal norms, these processes tend to render boys' roles in perpetrating bullying episodes invisible while highlighting girls' roles as problematic (Mishna et al., 2020b).

While the prevailing portrayal of boys across the participant narratives was that they are “chill” and easily forget situations, a few participants linked such attitudes and behaviors to dominant socialization expectations resulting in boys acting in a manner so as not to appear weak. Likewise, while participants overwhelmingly represented girls as escalating issues, a few participants suggested that such behaviors are due to girls' socialization processes through which they are raised to aspire to certain physical looks and to act in ways that make them appear vulnerable. Participants' overall narratives of boys' and girls' involvement in bullying is consistent with previous research findings whereby boys' bullying (e.g., aggression) was not considered bullying or was viewed as less harmful than girls' bullying (Ringrose and Renold, 2010).

While presenting evidence of a “chill” demeanor, participants frequently commented on boys engaging in punching, beating up or fighting and then quickly moving on or not dwelling on the interaction. Interpreting physical aggression in this manner suggests the normalization of physical aggression among boys, thereby enforcing hegemonic masculinity. This portrayal corresponds with the literature in which boys' behavior that reflects masculinity, such as bullying, is excused and accepted (Rosen and Nofziger, 2019). According to the participants in our study, further evidence of boys as “chill” was that they did not appear to be bothered when they themselves were bullied, in contrast to girls who were considered to be “overly sensitive.” Rosen and Nofziger (2019) posit that when boys who experience peer victimization indicate that they are not bothered, they are also confirming hegemonic masculinity. If boys were to acknowledge that they are being victimized, “they are admitting their vulnerability and defeat, thereby calling into question their masculinity” (Rosen and Nofziger, 2019, p. 312). Scholars argue that to understand bullying, the ecological systems framework must be elaborated to consider the influence of a patriarchal system (Felix and Greif Green, 2009; Garandeau et al., 2010; Mishna et al., 2020b). Framing bullying within an ecological systems framework that draws attention to patriarchal systems allows for a more fulsome understanding of the complex relationship dynamics in which bullying occurs. This approach will help to inform effective assessment, education, prevention, and intervention strategies.

### Type of Bullying—Comparing Traditional Bullying and Cyberbullying

Participants' assumptions regarding the type of bullying, its ease of perpetration and its severity and impact often precluded understanding the full context and relationship dynamics of bullying. For instance, the students, their parents and teachers typically considered cyberbullying easier to engage in than traditional bullying, due to the lack of visual cues from the victim in response to the bullying or direct contact. This view is consistent with the process of online disinhibition described in the literature, whereby individuals become less inhibited or less fearful of others' judgements in expressing their thoughts and feelings online, than they would be in face-to-face interactions (Huang and Chou, 2013; Lapidot-Lefler and Dolev-Cohen, 2015).

While participants believed that cyberbullying was easier, their corollary inference was that individuals' conscience and empathy would make persisting with traditional bullying more difficult. Accordingly, many participant narratives indicated that traditional bullying would be easier to stop and/or curtail. Research, however, does not support this belief and indeed, suggests that the frequency rather than the type of bullying may be related to the moral disengagement of perpetrators. Students who bully more often, either online or through traditional means, are less likely to report guilt and remorse in response to their bullying behaviors, thereby suggesting deeper moral disengagement compared to those who bully less frequently (Wachs, 2012). Moreover, despite parents identifying cyberbullying as their greatest fear, teenagers report that traditional bullying occurs more frequently than does cyberbullying (Ybarra et al., 2012). Nevertheless, Thornberg and Delby (2019) found that students' knowledge of the negative impact of bullying is eclipsed by the force of relationship dynamics and social processes: “Social rewards outclass moral concerns” (p. 150). Thus, the relationship dynamics, including the gendered behaviors and social rewards of bullying, may overpower individual characteristics such as empathy and guilt. To intervene effectively in youth's social processes, it is critical that adults recognize and understand the relationship dynamics in bullying such as power imbalances and the intent to hurt (Pepler et al., 2010).

### Physical Bullying as Disconnected From Relationship Dynamics

A striking finding is that many participants portrayed physical bullying as detached from relationship dynamics. When discussing physical bullying participants often expressed the view that the effects were “short-term” and would “heal quickly” in comparison to all other types of bullying which were understood to have lasting negative “mental” and psychological effects. This false dichotomy then tended to preclude participants from understanding physical bullying as occurring within complex relationship dynamics and was commonly associated with participants' gendered assumptions. In focusing on visible injuries associated with physical bullying, a number of parents and teachers as well as students both minimized the effects of the physical bullying and overlooked the relationship dynamics and associated harmful psychological and social effects of bullying.

This view of physical bullying as separate from relationships is not supported by the research literature that identifies *all* bullying as involving complex relationship dynamics (e.g., power imbalance, intent to hurt). For instance, Malhi et al. (2015) found that victims of physical bullying, most often boys, report more difficulties with peer relationships compared to those who are victims of relational bullying, demonstrating clear relationship effects for boys who experience physical bullying at the hands of peers. Perceiving bullying in terms of group processes and impacts provides greater understanding of motivations for bullying and factors that maintain it, as well as the inadequate support for victims (Salmivalli et al., 2010).

## Limitations

While this study draws from a large and diverse sample, there are limitations. The study did not include analysis and comparison of participant responses according to factors such as socioeconomic status, ethnicity and race, or children and youth's intersecting identities, such as youth who identify as gender nonconforming. While the sample was recruited from a large urban school board and thus the findings may not be relevant to other locales, our findings are consistent with the research literature.

## Conclusion

The express inclusion of student, parent and teacher perspectives represents three critical systems of relationships in the ecological context of bullying and provides an opportunity to address the social relationships and power dynamics that are fundamental to all bullying. Central to participants' understanding of traditional bullying and cyberbullying was whether they considered bullying to represent harmful relationship dynamics. Assumptions about gender and bullying shaped their understanding of bullying and precluded a conceptualization of bullying as involving complex relationship dynamics.

Close analysis of the interviews paradoxically revealed that while participants' narratives tended to overlook the relationship dynamics of bullying, they used terms that underscored these dynamics such as “*exclusion*,” “*intimidation,” “intentionality*,” and “*humiliation*.” As bullying repeatedly occurs, it exacerbates the power imbalance rendering the victimized student incapable of escaping these harmful relationships. It is essential, therefore, that other students, parents, and teachers recognize the inherent relationships and consistently act to stop the bullying and ensure that victimized students are safe and included. Such intervention can only be carried out with an understanding of the complex nature of bullying and of the social dynamics that maintain bullying and favor those with power (Smit, 2018). To counteract the diffusion of responsibility in addressing bullying, assessment, education, and intervention strategies in traditional and cyberbullying must be implemented at multiple levels of relationships and broader social systems, and “managed accordingly through relational leadership and an ethics of care” (Smit, 2018, p. S2).

While reflecting on traditional and cyberbullying during the interviews, some students, parents, and teachers shifted their views and understanding, which corresponds with research findings that information can affect how individuals respond (Kallestad and Olweus, 2003; Mishna et al., 2006). This unanticipated finding highlights the need to provide sensitive assessment, education and prevention and intervention strategies that focus on the complex relationship dynamics in bullying, and to challenge assumptions to the contrary.

## Data Availability Statement

The datasets presented in this article are not readily available because they must fit the restrictions of the Research Ethics Board. Requests to access the datasets should be directed to Faye Mishna (f.mishna@utoronto.ca).

## Ethics Statement

The studies involving human participants were reviewed and approved by the University of Toronto Health Sciences Research Ethics Board and the School Board External Research Review Committee. Written informed consent for students to participate in this study was provided by the participants' legal guardian/next of kin.

## Author Contributions

All authors listed have made a substantial, direct and intellectual contribution to the work and approved it for publication.

## Conflict of Interest

The authors declare that the research was conducted in the absence of any commercial or financial relationships that could be construed as a potential conflict of interest.
